# The Effect of Food Hardness on the Development of Dental Caries in Alloxan-Induced Diabetic Rats

**DOI:** 10.1155/2013/787084

**Published:** 2013-05-22

**Authors:** Yutaka Nakahara, Tomoya Sano, Yasushi Kodama, Kiyokazu Ozaki, Tetsuro Matsuura

**Affiliations:** ^1^Department of Pathology, Faculty of Pharmaceutical Sciences, Setsunan University, Osaka 573-0101, Japan; ^2^Laboratory of Clinicopathological Therapeutics, Faculty of Pharmaceutical Sciences, Hiroshima International University, Hiroshima 737-0112, Japan

## Abstract

We have previously shown that dental caries may be produced in diabetic rodent models fed with noncariogenic standard diets; however, many studies usually add large amounts of sugar to the diet to induce dental caries. Moreover, the physical properties of cariogenic diets have been reported as an important factor in the formation of caries. The aim of this study was to clarify the effect of the hardness of non-cariogenic diets on the development of dental caries in diabetic rodents. Seven-week-old female F344 rats were divided into 4 groups: intact rats fed with a standard pelletized or powdered diet and alloxan-induced diabetic rats fed with a standard pelletized or powdered diet. All of the rats were sacrificed at 52 weeks of age for morphological examinations on their dental tissue. Dental caries had developed and extended to all the molars in the diabetic rats that were fed with both the pelletized and powdered diets. Moreover, the lesion was significantly enhanced in the powdered diet group compared to that in the pelletized diet group. In conclusion, food hardness is an important factor influencing the development of dental caries in diabetic rats.

## 1. Introduction

We recently demonstrated that diabetes directly induced dental caries in rats and mice, although a direct association between the 2 was less evident and conflicting in humans [1–4]. We demonstrated that dental caries were produced using noncariogenic diets with a low concentration of sugar in diabetic rodent models [5–7], although many studies usually add large amounts of sugar to the diet to induce dental caries [8, 9].

In addition, early studies on the production of experimental dental caries have reported the effects of physical properties of cariogenic diets with high concentrations of sugar on dental caries. These studies mainly used finely ground cariogenic diet [10–13] because it is more cariogenic compared to a coarsely ground diet in rodents. Moreover, hard and coarse foods have the ability to prevent caries [14–17]. Thus, modifying the dietary formulation may possibly enhance or reduce caries development in diabetic animals. However, no reports have investigated the effects of the physical properties of non-cariogenic standard diets on dental caries in diabetic animals. 

Currently, 2 types of non-cariogenic standard diet formulations (i.e., powdered and pelletized diets) are widely used in experimental rodents. In the present study, we compared the effects of these diets on the development of dental caries in a diabetic rodent model focusing on the difference in hardness between the powdered and pelletized diets.

## 2. Materials and Methods

### 2.1. Animals and Housing Conditions

Six-week-old female F344 rats were supplied by Japan SLC, Inc. (Hamamatsu, Japan). The animals were housed in stainless-steel cages at a temperature of 20–26°C and a relative humidity of 40–70% under a 12/12 h light/dark cycle, that were ventilated with filtered fresh air. To prevent infection, the cages were changed at least once a week. The animals were allowed free access to tap water and fed with a widely used standard pelletized or powdered diet (Charles River Formula 1 (CRF-1); Oriental Yeast Co., Ltd., Tokyo, Japan) for experimental mice and rats. The animals were handled according to the principles for all experimental procedures outlined in the Guide for the Care and Use of Laboratory Animals prepared by our institution (Setsunan University) and the Japanese Association for Laboratory Animal Science.

### 2.2. Glucosuria and Glycemia Monitoring

Fresh urine samples were collected from the animals by using metabolism cages. The glucose levels in the fresh urine were measured semiquantitatively using urine test paper (Wako Pure Chemical Industries, Osaka, Japan) daily from day 1 to day 3 after dosing, once every week for 1 month after the first week, and once every month thereafter. The blood glucose levels in the tail vein samples were also measured semi-quantitatively using the glucose oxidase method (Glutest E; Sanwakagaku, Aichi, Japan) once every month from the fourth week after alloxan injection. Blood samples obtained from the tail vein and fresh urine were collected from 1:00 to 4:00 p.m. The severity of hyperglycemia was defined as follows: normal, <200 mg/dL; mild, >200 mg/dL; moderate, >300 mg/dL; or severe, >400 mg/dL [18]. The severity of glycosuria was defined as follows: normal, <100 mg/dL; mild, >100 mg/dL; moderate, >250 mg/dL; or severe, >500 mg/dL.

### 2.3. Experimental Design

**Figure 1:**
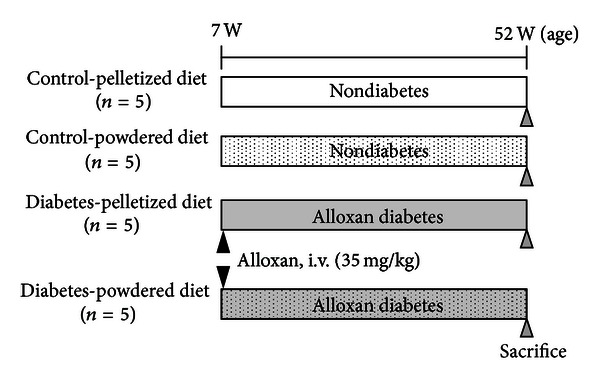
Study design.

The experimental design is shown in Figure 1. A total of 20 rats were randomly divided into 4 groups. Ten rats, aged 7 weeks, were given a single dose (35 mg/kg body weight) of alloxan (Sigma-Aldrich Japan, Tokyo, Japan) via tail vein injection. Alloxan, a pancreatic *β*-cell cytotoxic agent, was used to induce diabetes. The dose of alloxan was determined as the given dose at which a rat survives for a long period after the onset of diabetic symptoms. After the confirmation of hyperglycemia and glucosuria following the dosing of alloxan, the 10 rats were divided into 2 groups. Five rats were given a pelletized CRF-1 diet (diabetes-pelletized diet group) and the remaining 5 rats were given a powdered CRF-1 diet (diabetes-powdered diet group). As a control, each of the 5 intact rats was fed a pelletized or powdered CRF-1 diet (control-pelletized diet group and control-powdered diet group). All of the rats were sacrificed at 52 weeks of age for morphological examination.

### 2.4. Grading for Caries by Soft X-Ray Examination

The animals were euthanized by exsanguination under deep anesthesia at the end of the observation period. Subsequently, the mandible was removed and fixed in 10% neutral-buffered formalin (pH 7.4). After a 24 h fixation, the occlusal, buccolingual, and proximal surfaces of all of the molar teeth were intensively observed under a binocular stereoscope. Following macroscopic observation, a soft X-ray examination was performed. Soft X-ray images of the mesiodistal plane were taken under conditions of 35 kV and 2 mA for 4 min. The molar teeth were classified into 5 groups according to caries characteristics by observing and analyzing the radiographs: no radiolucent change (grade 0), radiolucent area only on the occlusal surface of the crown (grade 1), radiolucent areas on occlusal surface and either of the mesiodistal surfaces of the crown (grade 2), radiolucent areas over the entire surface of the crown (grade 3), and radiolucent areas over most of the surface of the dental root (grade 4). The mean score of the caries was used as indicator for comparing the severity of the carious lesions between the groups.

### 2.5. Histopathological Examination

After soft X-ray examination, a histopathological examination was performed on the mandible in all of the rats. After fixation with 10% neutral-buffered formalin, the sample was decalcified in a 5% solution of ethylenediaminetetraacetic acid 4 Na (EDTA 4 Na) for 2 weeks at 4°C. After decalcification, the specimens were trimmed, dehydrated in a sequential ethanol series using an automated processor, and embedded in paraffin wax. Serial 7 *μ*m thick sections on the mesiodistal plane were made through the centers of all of the molars and then stained with hematoxylin and eosin for examination using light microscopy. The severity of the caries lesion was graded as follows: slight, dentin caries localized in occlusal surface of dentin; mild, dentin caries extended into the dental pulp with pulpitis and/or pulp necrosis; moderate, dental crowns were partially decayed; and severe, dental crowns were completely decayed (only the molar roots remained).

### 2.6. Statistical Analysis

The Wilcoxon rank-sum test was employed to compare the differences in the mean scores of the caries lesions using soft X-ray examination between the groups. The chi-square test was used to determine the caries incidence using soft X-ray examination and the incidence of histopathological lesions in each group of rats. A *P* value of less than 0.05 was regarded as statistically significant.

## 3. Results

### 3.1. Blood and Urine Glucose Levels

**Figure 2:**
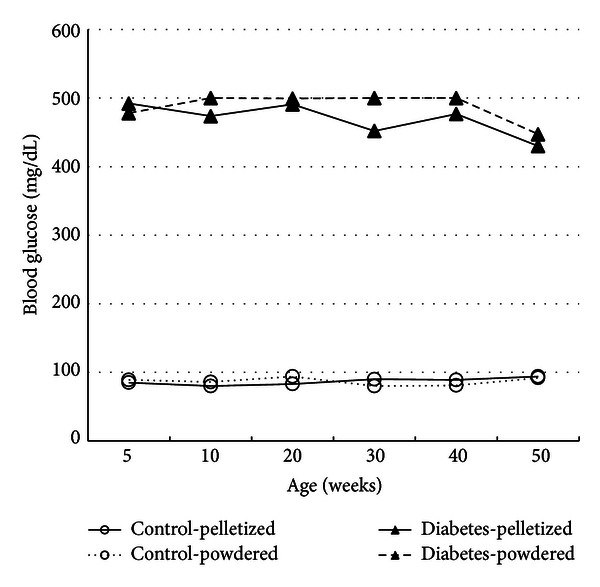
The mean blood glucose levels of each group. Severe hyperglycemia (>400 mg/dL) continued from the day after alloxan dosing to the time of the scheduled necropsy in all of the alloxan-treated F344 rats. Control F344 rats showed normal glycemia (<100 mg/dL).

The mean blood glucose levels of each group are shown in Figure 2. Severe hyperglycemia (>400 mg/dL) and glucosuria (>500 mg/dL) continued from the day after alloxan injection to the last monitoring day in all of the rats in the alloxan-treated groups. In addition, the blood glucose levels ranged from 78 to 120 mg/dL (normoglycemia), and the urine glucose levels were less than 100 mg/dL in the control groups. 

### 3.2. Morphological Characteristics of Carious Lesions

**Figure 3:**
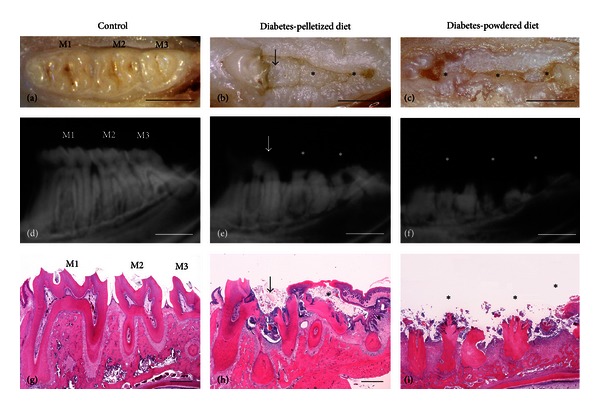
Morphological characteristics of molar caries in the mandibles of female F344 rats. M1: the first molar, M2: the second molar, and M3: the third molar. (a–c) Macroscopic appearance of molar caries. (a) Normal molars. (b) Partial coronal defect (arrow) of M1 and complete coronal defects (asterisks) of M2 and M3. (c) Complete coronal defects (asterisks) of all of the molars (M1–M3). Scale bar = 2 mm. (d–f) Soft X-ray images of the molar caries. (d) Normal molars. (e) Corresponding to the macroscopic observation, dental caries with a focal (arrow) or an extensive radiolucent area (asterisks) in the dental crown is observed. The caries were graded as 2 (M1), 4 (M2), and 3 (M3). (f) The dental crowns are completely absent in all of the molars (grade 4, asterisks). Scale bar = 2 mm. (g–i) Histopathological features of the carious lesions. (g) Normal molars. (h) Moderate-to-severe carious lesions. Dentin caries spread over a large part of the dental crown. (i) Severe carious lesions. Dentin caries expands to the dental root, resulting in a completely decayed crown. HE stain. Scale bar = 500 *μ*m.

Typical macroscopic appearances of carious molars in diabetic rats are shown in Figures 3(a), 3(b), and 3(c). Macroscopically, the dental caries developed mainly in occlusal fissures and were identified as partial coronal defects of the molars in alloxan-treated diabetic rats (Figure 3(b)). The carious lesions expanded horizontally, until the crown of the carious molar was completely invisible (Figures 3(b) and 3(c)). In contrast, control nondiabetic rats showed no changes in any of the molars (Figure 3(a)). 

**Figure 4:**
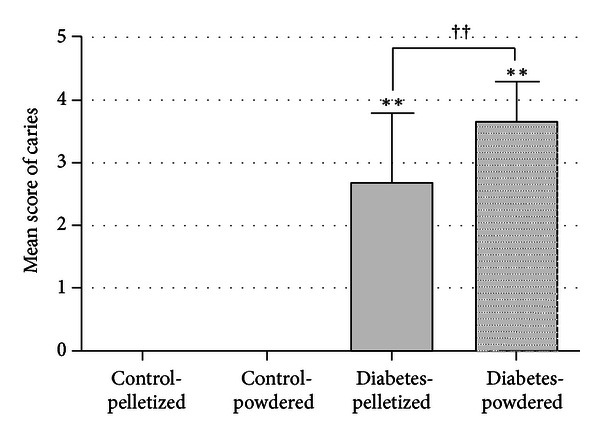
The mean scores for caries in the mandibular molars of each group. Significant difference from the control group (**P < 0.01) and from the diabetes-pelletized diet group (^††^ *P* < 0.01).

Soft X-ray images of the carious lesion in diabetic rats are shown in Figures 3(d), 3(e), and 3(f). In all of the alloxan-treated diabetic rats, dental caries were detected as radiolucent lesions in the dental crown. Radiographically, the dental caries progressed both horizontally and vertically. In diabetic rats fed with a pelletized diet, the carious lesion was mainly characterized as grade 2 (Figure 3(e)) or 4 type (Table 1). In addition, the diabetic rats fed a powdered diet demonstrated carious lesions that were nearly of grade 4 type (Figure 3(f)). In the control non-diabetic rats, there was no change in the molar teeth (Table 1, Figure 3(d)). 

The histopathological characteristics of the carious lesion in diabetic rats are shown in Figures 3(g), 3(h), and 3(i). Histopathological carious lesions were detected in the crown as eroded dentin with bacterial colonization in alloxan-treated diabetic rats. In diabetic rats fed with the pelletized diet, many of the molars were moderately affected and the dentin caries spread over a wide area of the dental crown (Figure 3(h)). In the diabetic rats fed with the powdered diet, the dental caries were markedly worsened and the crowns were nearly completely decayed (Figure 3(i)). 

### 3.3. Caries Incidence and Severity as Assessed Using Soft X-Ray and Histopathological Examination

The incidence of caries teeth on the basis of each scoring in the soft X-ray examination is shown in Table 1. The mean caries score of each group is shown in Figure 4. The incidence of dental caries in the diabetic rats fed on both the pelletized and powdered diets was apparently higher (*P* < 0.01) compared to the non-diabetic control rats (Table 1). In the diabetic rats fed with the pelletized diet, 96.7% of their molar teeth were affected with dental caries, and the mean caries score was 2.7. In addition, in the diabetic rats fed with the powdered diet, all of their molars (100%) were affected with caries, and the mean caries score was 3.7, which was significantly higher (*P* < 0.01) than the diabetic rats fed with the pelletized diet (Table 1, Figure 4). No radiolucent lesions were observed in any of the molars in control non-diabetic rats.

The incidence of histopathological carious lesions on the basis of each grade is summarized in Table 2. Histopathologically, the severity of the carious lesions in the diabetic rats fed with a powdered diet was significantly enhanced compared to the diabetic rats fed with the pelletized diet (Table 2). In control non-diabetic rats, slight carious lesions, which were macroscopically and radiographically normal, were detected in a few animals; however, there were no differences observed between the non-diabetic rats on the pelletized and powdered diets (Table 2).

## 4. Discussion

The physical properties of foods such as hardness, adhesiveness, and cohesiveness are closely related with the caries-producing potential [19, 20]. Moreover, the development of caries is known to be profoundly affected by food hardness, and hard and coarse foods can exert a detergent activity during mastication, which is effective in the prevention of caries [17, 19]. Furthermore, a lower degree of caries was reported in animals fed with the hardest food diet [16]. In the present study, the severity of caries in diabetic rats was enhanced in the powdered diet group compared to the pelletized diet group. It was clear that food hardness affected the development of dental caries in diabetic rats fed on a non-cariogenic diet. Hard foods are known to help flush away or neutralize undesirable material within the dental plaque and exert a cleaning effect on smooth surfaces by direct mechanical friction [17]. Therefore, cariogenicity will be higher in diabetic rats fed with a powdered diet than in those fed with a pelletized diet.

In this study, a high incidence and severity of dental caries was confirmed in the alloxan-treated diabetic rats fed with 2 types of standard diets with a low concentration of sugar. These results were consistent with our previous studies [5–7]. Furthermore, the mean caries score of the diabetic rats fed on the pelletized diet at 52 weeks after alloxan dosing in this study was almost 2-fold compared to animals at 26 weeks after alloxan dosing described in a previous report [7], strongly suggesting that the duration of hyperglycemia may affect caries development in diabetic animals. In this study, dental caries developed and extended to all of the molars in diabetic rats fed on both the pelletized and powdered diets. Thus, the effect of food hardness on the different diets in caries development in diabetic rats may become clearer when the dental caries mildly develop during the more early stages.

In conclusion, food hardness could have an effect on the development of dental caries in diabetic rats, and appropriate consideration of the food formulation should be made in experimental caries studies using diabetic rodent models. 

## Figures and Tables

**Table 1:** Incidence and grading of the carious molars in the mandibles of diabetic rats fed on a pelletized or powdered diet.

	Control (nondiabetic)		Diabetic	
Groups	Pelletized diet	Powdered diet	Pelletized diet	Powdered diet
	(*n* = 5)	(*n* = 5)	(*n* = 5)	(*n* = 5)
Number examined	30	30	30	30
Number of carious molars	0	0	29 (96.7%)**	30 (100%)^∗∗††^

Grade 0	30 (100%)	30 (100%)	1 (3.3%)	0
Grade 1	0	0	1 (3.3%)	0
Grade 2	0	0	14 (46.7%)	2 (6.7%)
Grade 3	0	0	4 (13.3%)	6 (20.0%)
Grade 4	0	0	10 (33.3%)	22 (73.3%)

Significant difference from the control group (**P < 0.01).

Significant difference from the diabetes-pelletized diet group (^††^ *P* < 0.01).

**Table 2:** Histopathological carious lesions in the mandibular molars of diabetic rats fed on a pelletized or powdered diet.

	Control (nondiabetic)		Diabetic	
Groups	Pelletized diet	Powdered diet	Pelletized diet	Powdered diet
	(*n* = 5)	(*n* = 5)	(*n* = 5)	(*n* = 5)
Carious lesion	1/30 (3.3%)	2/30 (6.7%)	30/30 (100%)**	30/30 (100%)^∗∗††^
Slight	1 (3.3%)	2 (6.7%)	2 (6.7%)	0
Mild	0	0	8 (26.7%)	2 (6.7%)
Moderate	0	0	13 (43.3%)	5 (16.7%)
Severe	0	0	7 (23.3%)	23 (76.7%)

Significant difference from the control group (**P < 0.01).

Significant difference from the diabetes-pelletized diet group (^††^ *P* < 0.01).
